# The Conserved ESCRT-III Machinery Participates in the Phagocytosis of *Entamoeba histolytica*

**DOI:** 10.3389/fcimb.2018.00053

**Published:** 2018-03-01

**Authors:** Yunuen Avalos-Padilla, Roland L. Knorr, Rosario Javier-Reyna, Guillermina García-Rivera, Reinhard Lipowsky, Rumiana Dimova, Esther Orozco

**Affiliations:** ^1^Department of Theory and Bio-Systems, Max Planck Institute of Colloids and Interfaces, Potsdam, Germany; ^2^Departamento de Infectómica y Patogénesis Molecular, CINVESTAV IPN, Mexico City, Mexico

**Keywords:** protozoan parasites, ESCRT-III proteins, *Entamoeba histolytica*, phagocytosis, GUVs model

## Abstract

The endosomal sorting complex required for transport (ESCRT) orchestrates cell membrane-remodeling mechanisms in eukaryotes, including endocytosis. However, ESCRT functions in phagocytosis (ingestion of ≥250 nm particles), has been poorly studied. In macrophages and amoebae, phagocytosis is required for cell nutrition and attack to other microorganisms and cells. In *Entamoeba histolytica*, the voracious protozoan responsible for human amoebiasis, phagocytosis is a land mark of virulence. Here, we have investigated the role of ESCRT-III in the phagocytosis of *E. histolytica*, using mutant trophozoites, recombinant proteins (rEhVps20, rEhVps32, rEhVps24, and rEhVps2) and giant unilamellar vesicles (GUVs). Confocal images displayed the four proteins located around the ingested erythrocytes, in erythrocytes-containing phagosomes and in multivesicular bodies. EhVps32 and EhVps2 proteins co-localized at the phagocytic cups. Protein association increased during phagocytosis. Immunoprecipitation and flow cytometry assays substantiated these associations. GUVs revealed that the protein assembly sequence is essential to form intraluminal vesicles (ILVs). First, the active rEhVps20 bound to membranes and recruited rEhVps32, promoting membrane invaginations. rEhVps24 allowed the detachment of nascent vesicles, forming ILVs; and rEhVps2 modulated their size. The knock down of *Ehvps20* and *Ehvps24*genes diminished the rate of erythrophagocytosis demonstrating the importance of ESCRT-III in this event. In conclusion, we present here evidence of the ESCRT-III participation in phagocytosis and delimitate the putative function of proteins, according to the *in vitro* reconstruction of their assembling.

## Introduction

In eukaryotic cells, endocytosis is the universal process to ingest nutrients and separate proteins for digestion and recycling pathways. The endosomal sorting complex required for transport (ESCRT) orchestrates several important cell membrane-remodeling mechanisms. Endocytosis involves the ingestion of small particles (≤250 nm) and of large particles (≥250 nm), including cells. In the latter case, one usually speaks of phagocytosis, a mechanism also used as an attack strategy against bacteria and other target cells. Even though the ingestion of small and large particles has many common features, phagocytosis involves a more complex molecular machinery (including contractil actin myosin ring).

One important aspect of phagocytosis is that it is a key event in the virulence mechanism of *Entamoeba histolytica*, the protozoan cause of human amoebiasis, responsible for killing 100,000 people each year (Mortimer and Chadee, 2010). Some of the molecules involved in the phagocytosis of *E. histolytica* have been already discovered. They are localized in the plasma membrane, in phagocytic cups (Arroyo and Orozco, 1987; Petri et al., 2002; Seigneur et al., 2005; Jain et al., 2008), and in the endosomes and internal membranes (Saito-Nakano et al., 2004; Loftus et al., 2005; Castellanos-Castro et al., 2016). Earlier, we identified the EhADH protein (an ALIX family member) that together with EhCP112 (a cysteine protease), forms the EhCPADH virulence complex (García-Rivera et al., 1999), which interacts with the Gal/Gal lectin at the trophozoites surface (Seigneur et al., 2005). EhADH possesses an adherence epitope at the C-terminus, which interacts with target cells (Arroyo and Orozco, 1987; García-Rivera et al., 1999) and a Bro1 domain at the N-terminus that faces the cytoplasm (Bañuelos et al., 2005). The EhADH protein binds to the EhVps32 protein (Bañuelos et al., 2012; Avalos-Padilla et al., 2015), which is the most abundant member of the ESCRT machinery.

In eukaryotes, ESCRT-0, ESCRT-I, ESCRT-II, ESCRT-III complexes and accessory proteins, among them ALIX and the Vps4 ATPase, form the ESCRT machinery (Babst et al., 2002a,b; Katzmann et al., 2002). ESCRT also participate in cytokinesis, virus budding and other cellular processes that require membrane fusion and fission (Strack et al., 2003; Morita et al., 2007; Hurley, 2015). During endocytosis, ESCRT-0 contacts the cargo and recruits ESCRT-I and ESCRT-II. Together, they trigger membrane invaginations in an opposite topology to that described in clathrin-coated cargo-carrying vesicles (Williams and Urbé, 2007). Then, ESCRT-II recruits ESCRT-III, whose proteins oligomerise on the endosomal membrane, stretching the preformed necks and generating intraluminal vesicles (ILVs) in the multivesicular bodies (MVBs). Later, Vps4 ATPase disassembles the ESCRT-III complex and the proteins return to the cytoplasm to start a new ILVs formation round (Babst et al., 1998, 2002a).

*E. histolytica* possesses the majority of the ESCRT machinery genes (López-Reyes et al., 2011), but their role in phagocytosis is poorly understood. To further elucidate the phagocytosis puzzle, we performed *in vivo* and *in vitro* studies using mutant trophozoites, the *E. histolytica* ESCRT-III purified recombinant proteins (rEhVps20, rEhVps32, rEhVps24, and rEhVps2) and giant unilamellar vesicles (GUVs). Our results demonstrated that in trophozoites, ESCRT-III proteins are present in the phagocytic cups and then, surround the ingested erythrocytes. Later, they appear in the erythrocyte-containing phagosomes and MVB-like structures. In GUVs, rEhVps20 bound to the membrane and recruited rEhVps32 which promoted the formation of quasi-spherical buds connected to the membranes by narrow necks. rEhVps24 cleaved the buds provoking the formation of ILVs; whose size was modulated by rEhVps2. The knock down of *Ehvps20* and *EhVps24* genes diminished the rate of phagocytosis, providing evidence for the importance of the ESCRT machinery in this process.

## Results

### *E. histolytica* possesses the four orthologs of the ESCRT-III complex

Earlier, we identified and partially characterized the *Ehvps2, Ehvps24*, and *Ehvps32* genes and transcripts, as well as the EhVps32 protein, members of the ESCRT-III complex (López-Reyes et al., 2011; Avalos-Padilla et al., 2015). However, there are no studies regarding the *Ehvps20* gene and its product and we have not dissected the proteins function. Here, we first detected and cloned the *Ehvps20* gene and then, produced the four ESCRT-III recombinant proteins to elucidate their putative function.

**Figure 1:**
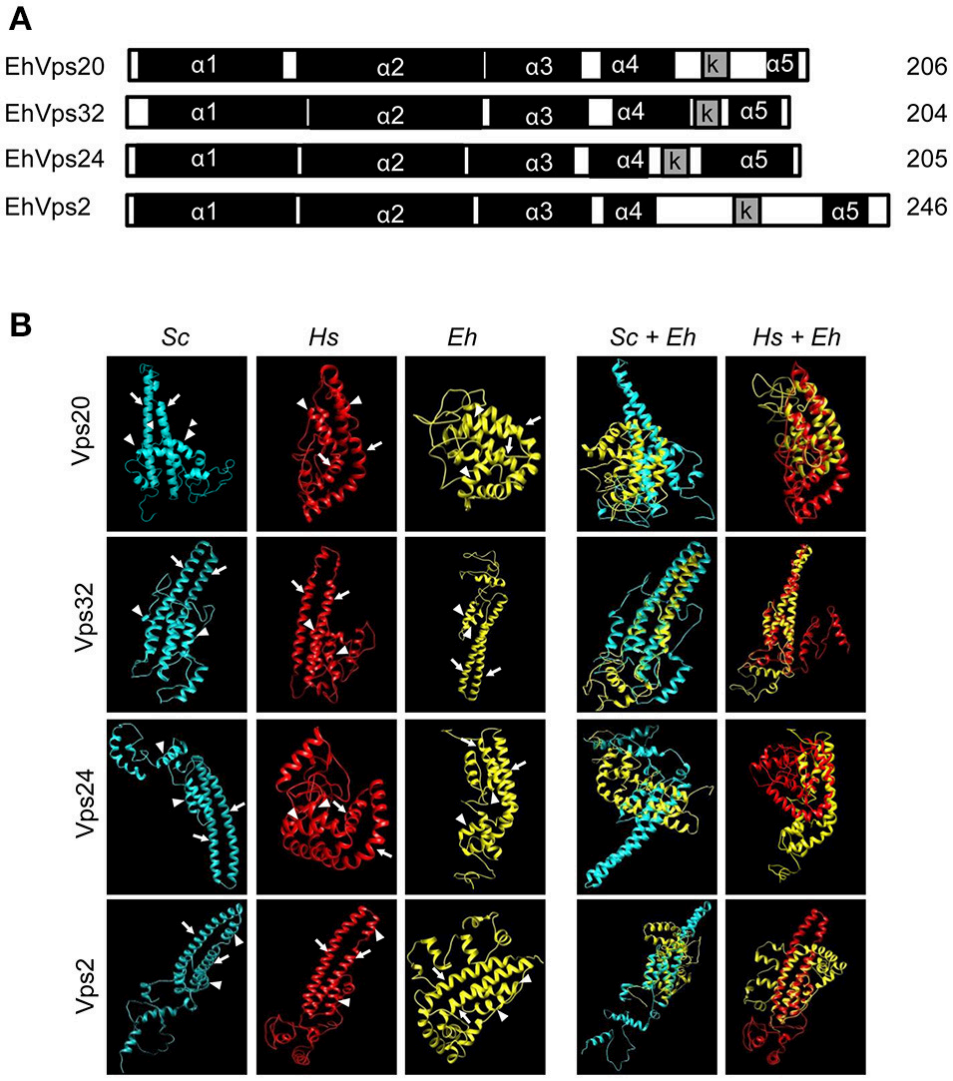
*E. histolytica* ESCRT-III proteins present the same structure of their orthologs. **(A)** Schematic representation of the four ESCRT-III proteins. Black squares: α-helixes (α1, α2, α3, α4, and α5), gray squares: k-linker (*k*). Numbers at right: amino acid number of each protein. **(B)** 3D structures of ESCRT-III proteins from yeast (*Sc*), human (*Hs*), and *E. histolytica* (*Eh*). Right panels: overlapping of 3D structures of yeast or human and *E. histolytica* proteins. Arrows show the two long α-helices and arrowheads the two short α-helices in the core domain.

We employed the SNF7 domain sequence, present in all ESCRT-III homologs (Winter and Hauser, 2006) to search for the *Ehvps20* gene in the AmoebaDB (http://amoebadb.org/amoeba/). By this domain, we found the EHI_114790 sequence with a 621 bp open reading frame carrying a 53 bp intron, and predicting a 206 amino acids protein (Figure 1A). This protein hereafter referred as EhVps20, has 45% homology to the *Saccharomyces cerevisiae Scvps20* gene, 36% to the *Homo sapiens Chmp6* gene and 23% identity to both genes.

The four putative *E. histolytica* ESCRT-III genes predicted proteins of 204–246 amino acids (Figure 1A). As their orthologs, proteins exhibited positively charged α-helices in the N-terminal domains, and negatively charged α-helices in the C-termini (Babst et al., 2002a). Secondary structures of all proteins presented the highly hydrophobic “k-linker” sequence, located between the fourth and fifth α-helices (Figure 1A). The k-linker is essential for protein opening and closing to generate the protein active and inactive states, respectively (Henne et al., 2012). Comparison of the predicted *E. histolytica* 3D structures (retrieved from the *Phyre2* server) with the *S. cerevisiae* and *H. sapiens* orthologs exposed in all proteins, a core domain formed by two long and two short α-helices (Figure 1B, arrows and arrowheads, respectively), like the unveiled ESCRT-III homolog crystals (Muzioł et al., 2006; Bajorek et al., 2009; Martinelli et al., 2012). Judging by their 3D structures, *E. histolytica* ESCRT-III proteins are more similar to their human than to their yeast orthologs (Figure 1B). The low homology in sequence and structure of ESCRT-III orthologs (Table 1), together with the high homology in key domains, point out to the relevance of the SNF7 domain, α-helices and k-linkers in the protein function.

**Table 1:** Percentage of identity and similarity and predicted functions of ESCRT-III proteins.

***Entamoeba histolytica***		***Homo sapiens***					***Saccharomyces cerevisiae***					**Function**
**Predicted protein**	**Accession number**	**Protein**	**Accession number**	***E*-value**	**S (%)**	**I (%)**	**Protein**	**Accession number**	***E*-value**	**S (%)**	**I (%)**	
EhVps2	C4LZV3	CHMP2A	Q43633	2.00E-32	51	32	Vps2	P36108	3.00E-22	43	28	Participate in the scission of the ILV and recruits Vps4 ATPase
		CHMP2B	Q9UQN3	4.00E-22	40	27						
EhVps20	C4M7T5	CHMP6	Q96FZ7	8.00E-08	36	23	Vps20	Q04272	0.008	45	23	Binds to ESCRT-II and recruits Vps32
EhVps24	C4M2Y2	CHMP3	Q9Y3E7	0.035	20	22	Vps24	P36095	0.07	20	25	Participate in the scission of the ILV and recruits Vps4 ATPase
EhVps32	C4M1A5	CHMP4A	Q9BY43	1.00E-10	40	29	Vps32	P39929	4.00E-12	39	27	Binds to EhVps20 and forms helical polimers that close the neck of the nascent ILV
		CHMP4B	Q9H444	7.00E-08	41	27						
		CHMP4C	Q96CF2	2.00E-06	39	24						

*The e-value and the percentages of identity and similarity were obtained by comparing the sequences of E. histolytica ESCRT-III putative proteins with their orthologs in Human and Yeast*.

#### ESCRT-III proteins participate in the erythrophagocytosis of *E. histolytica*

In yeast and mammals, the inactive forms of ESCRT-III proteins are soluble in the cytoplasm; whereas in their active state, they bind to the endosomal membrane to promote the continuity of endocytosis until cargo digestion and protein recycling occur (Shim et al., 2007). Given its participation in receptor-mediated endocytosis, the ESCRT machinery is candidate to have a central role in phagocytosis. Nevertheless, molecular mechanisms in both events could differ, mainly due to the size and complexity of the ingested cargo.

**Figure 2:**
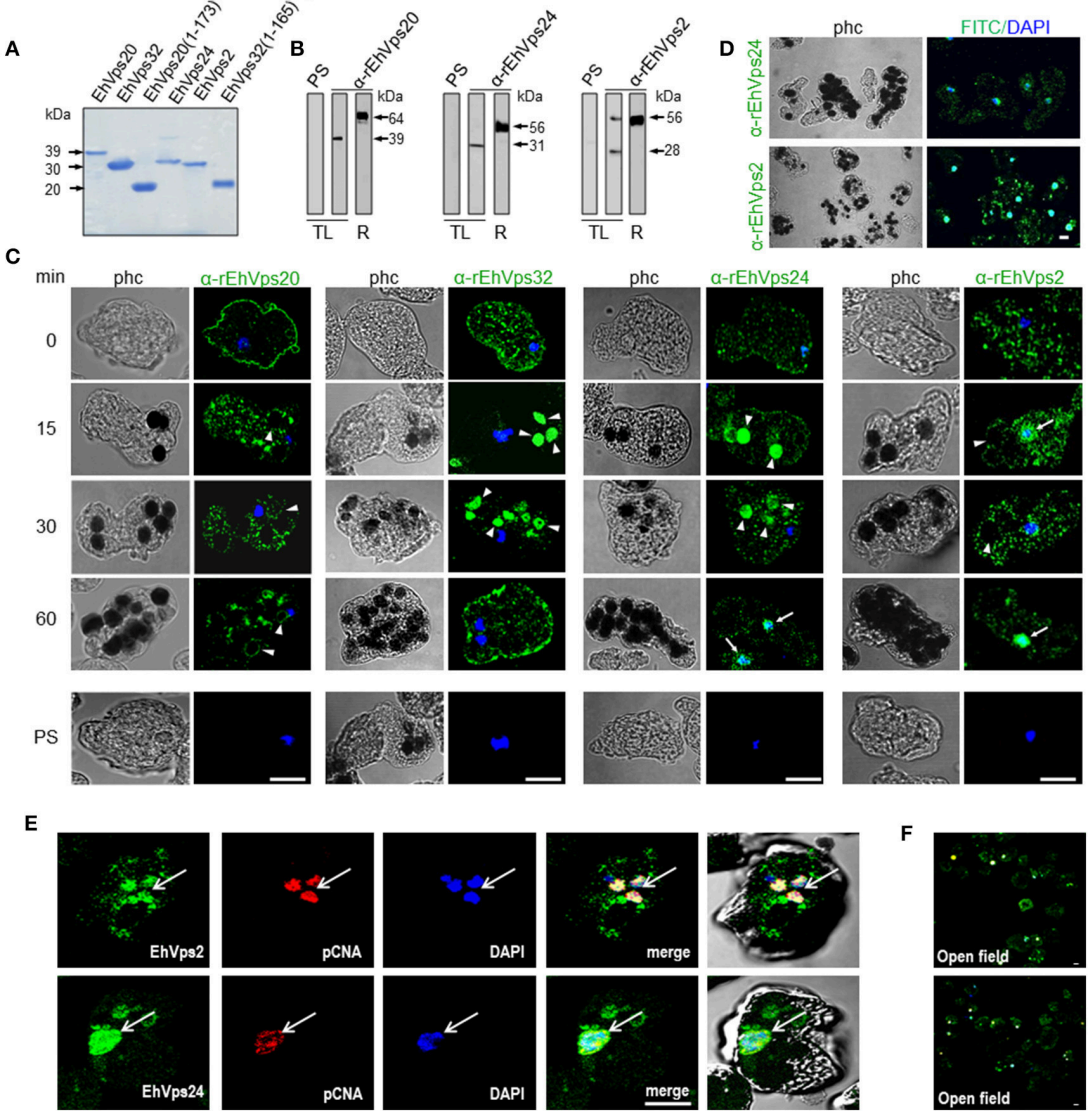
ESCRT-III proteins are located in cytoplasm, vesicles, nuclei and around phagosomes of trophozoites. **(A)** Coomassie blue stained polyacrylamide gel of the recombinant proteins (without GST-tag). **(B)** Western blot of trophozoites lysates (TL) and purified GST-tagged recombinant proteins (R) using specific antibodies or pre-immune sera (PS). **(C)** Representative images of 4% paraformaldehyde fixed trophozoites treated with the anti-ESCRT-III proteins antibodies (green) in basal conditions (0 min) and during erythrophagocytosis. Arrowheads show the antibodies signal in erythrophagosomes. Arrows shows the presence of the proteins in the nuclei stained by DAPI. PS: pre-immune serum. **(D)** Open field of trophozoites stained by α-rEhVps24 or α-rEhVps2 and corresponding secondary antibodies and DAPI after 60 min of erythrophagocytosis. **(E)** Representative images of 4% paraformaldehyde fixed trophozoites treated with the αEhVps24 or α-rEhVps2 (green) and α-pCNA protein (red) antibodies after 60 min of erythrophagocytosis. Nuclei were stained by DAPI. **(F)** Open field of trophozoites stained by αEhVps24 or α-rEhVps2 and α-pCNA with corresponding secondary antibodies after 60 min of erythrophagocytosis. phc, phase contrast. Scale bar: 10 μm.

To study the role of the ESCRT-III in phagocytosis, we cloned the genes, expressed their products and generate specific antibodies against each protein. Coomassie blue stained gels confirmed the integrity of the proteins by their predicted molecular weight (Figure 2A). By western blot assays, the specific antibodies recognized the rEhVps20 and rEhVps24 recombinant proteins and a single band in trophozoite lysates (Figure 2B). Similarly to the EhVps32 protein, EhVps20 and EhVps24 consistently exhibited higher molecular weight (39 and 31 kDa, respectively) than the predicted ones by the amino acid sequence (24 kDa for both) (Figure 2B) (Avalos-Padilla et al., 2015). However, bacterially expressed EhVps20 and EhVps24 also migrated in an identical manner. As in their orthologs, the highly charged nature of the proteins could influence their migration (Babst et al., 2002a). Moreover, when we removed the last alpha helix from EhVps20 and EhVps32, where the acidic charges are concentrated, the recombinant proteins migrated at the expected molecular weight. This supports our hypothesis for the observed discrepancy of the molecular weights. In contrast, rEhVps2 migrated at its predicted molecular weight (28 kDa), but in trophozoite lysates, α-rEhVps2 antibody revealed 28 and 56 kDa bands. This last could be a dimer or a complex formed by EhVps2 with another unidentified protein (Figure 2B) but more experiments are need to prove this. Pre-immune serum did not react with any protein from bacterial or trophozoites lysates.

To localize the proteins in the cell, we performed confocal microscopy assays of trophozoites in basal conditions (0 time, no phagocytosis) and after phagocytosis. FITC-labeled secondary antibodies detected each one of the specific antibodies and proteins. In basal conditions, EhVps20, EhVps32, EhVps24, and EhVps2 appeared in punctuated structures dispersed in the cytoplasm. EhVps20, EhVps32, and EhVps24 localized adjacent to plasma membrane (Figure 2C). As the antibodies gave no signal in non-permeabilized trophozoites, we concluded that like EhVps32 (Avalos-Padilla et al., 2015), EhVps20 and EhVps24 were close to the inner leaflet of the plasma membrane.

After 15–30 min of phagocytosis, all four proteins appeared spread in the cytoplasm and in the membranes and lumen of phagosomes and surrounding the ingested erythrocytes (Figure 2C). Intriguingly, EhVps24 and EhVps2, consistently migrated to the nuclei at longer times of phagocytosis (60 min), co-localizing with the nuclear protein pCNA (Trasviña-Arenas et al., 2017) and DAPI staining (Figures 2C,E). Altogether, these results demonstrated that the ESCRT-III orthologs associate to erythrocytes and erythrocyte-containing phagosomes, suggesting that ESCRT-III proteins are a part of the machinery involved in phagocytosis. Yet, we have no data to explain the presence of ESCRT-III proteins in the nucleus. We speculate that they could be regulating or co-regulating some unidentified nuclear function; but further experiments are necessary to prove this.

#### ESCRT-III proteins interact with each other during erythrophagocytosis

We explored the possible association among the ESCRT-III proteins in basal conditions and during erythrophagocytosis by immunofluorescence, immunoprecipitation, flow cytometry and *in vitro* assays using GUVs.

**Figure 3:**
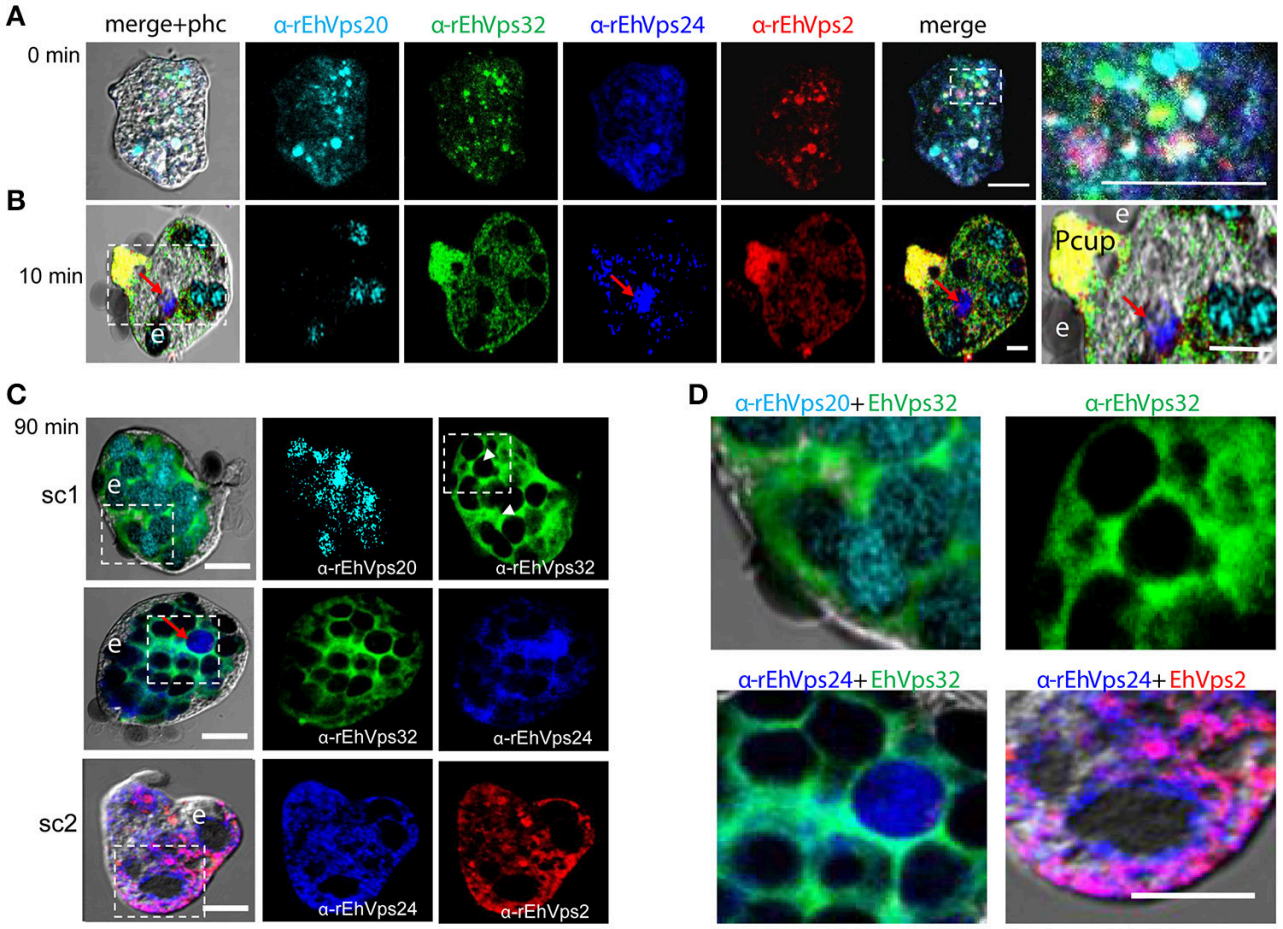
ESCRT-III proteins co-localize during phagocytosis of *E. histolytica*. **(A–D)** Representative confocal images of trophozoites in basal conditions and after different times of erythrophagocytosis. PFA-fixed trophozoites were incubated with FITC or TRITC labeled secondary antibodies after incubation with α-rEhVps32 (green) and α-rEhVps2 (red) primary antibodies, respectively; Alexa647 (cyan) and Pacific Blue (blue) directly labeled the α-rEhVps20 and α-rEhVps24 antibodies, respectively. **(A)** Basal conditions. At right: magnification of white square. **(B)** Trophozoites at 10 min of erythrophagocytosis. At right: magnification of white square. Pcup: phagocytic cup, e: erythrocytes, red arrow: vesicle stained only by α-rEhVps24 antibody. **(C)** Confocal representative image of a trophozoite after 90 min of phagocytosis. The areas are marked in squares are magnified in **(D)**: upper panels: erythrocytes covered by EhVps20 and surrounded by EhVps32 and EhVps32 covering two erythrocytes (e) (arrowhead in **C**) in contrast to others that are surrounded by the protein. Lower panel left: EhVPs32 forming the honeycomb like panel around the erythrocytes together with EhVps24. Lower panel right: EhVps2 and EhVps24 together surrounding the partially digested erythroctyes. phc, phase contrast; sc1, subcomplex 1; sc2, subcomplex 2. Scale bar: 5 μm.

In basal conditions, confocal images showed all ESCRT-III proteins dispersed in the cytoplasm as clumps, or inside vesicles of different size. Proteins were grouped, co-localizing in distinct combinations (Figure 3A, see magnification square of merging image). The observed vesicles with more than one protein may be due to the active constitutive endocytosis in trophozoites.

After 10 min of phagocytosis, EhVps20 appeared around the ingested erythrocytes and dispersed in the cytoplasm (Figure 3B, cyan). A part of EhVps32 remained in the plasma membrane and the cytoplasm, but another part also migrated to the area close to the protrusion forming the phagocytic cup and to the membrane of vesicles with ingested erythrocytes (Figure 3B, green). EhVps24 appeared in the vesicles with ingested erythrocytes (Figure 3, blue), co-localizing with other proteins, but frequently, it was alone in a vesicle (Figure 3B, red arrow). EhVps2 strongly marked the phagocytic cup and appeared in the cytoplasm around the ingested erythrocytes (Figure 3B, red). Surprisingly, in many trophozoites, EhVps32 and EhVps2 co-localized at the phagocytic cup to a higher extent than in phagosomes (Figure 3B, see magnification square, yellow).

Subsequently, we explored the localization of the ESCRT-III proteins in phagosomes after 90 min of erythrophagocytosis. At this time, hemoglobin is in an advanced digestion process, but is still possible to distinguish the remnant erythrocytes. For these experiments, we used couples of antibodies for the same preparation:α-EhVps20/α-EhVps32 (sc1), α-EhVps32/α-EhVps24, and α-EhVps24/α-EhVps2 (sc2). The accumulation of erythrocytes inside the trophozoites, produced phagosomes of distinct shapes and size, depending on the number of erythrocytes they carried and on the ingestion time. Confocal images showed that EhVps20 covered the phagosomes with a uniform punctuate pattern (Figure 3C, see magnification in 3D) and it co-localized in some points with EhVps32. EhVps32 defined a honeycomb-like arrangement around the erythrocytes inside the huge phagosomes (Figure 3C, see magnification in 3D upper, right panel). EhVps24 and EhVps2 appeared diffuse in the cytoplasm and around the phagosomes, co-localizing with each other and surrounding the erythrocytes. In many images, EhVps2 appeared closer to the erythrocytes (Figure 3C, see magnification in 3D, lower, right panel). These results exhibited the four ESCRT-III proteins in the erythrocyte-containing phagosomes, on the ingested erythrocytes and around them, but proteins displayed different patterns which varies according to the time of the erythrocytes ingestion.

**Figure 4:**
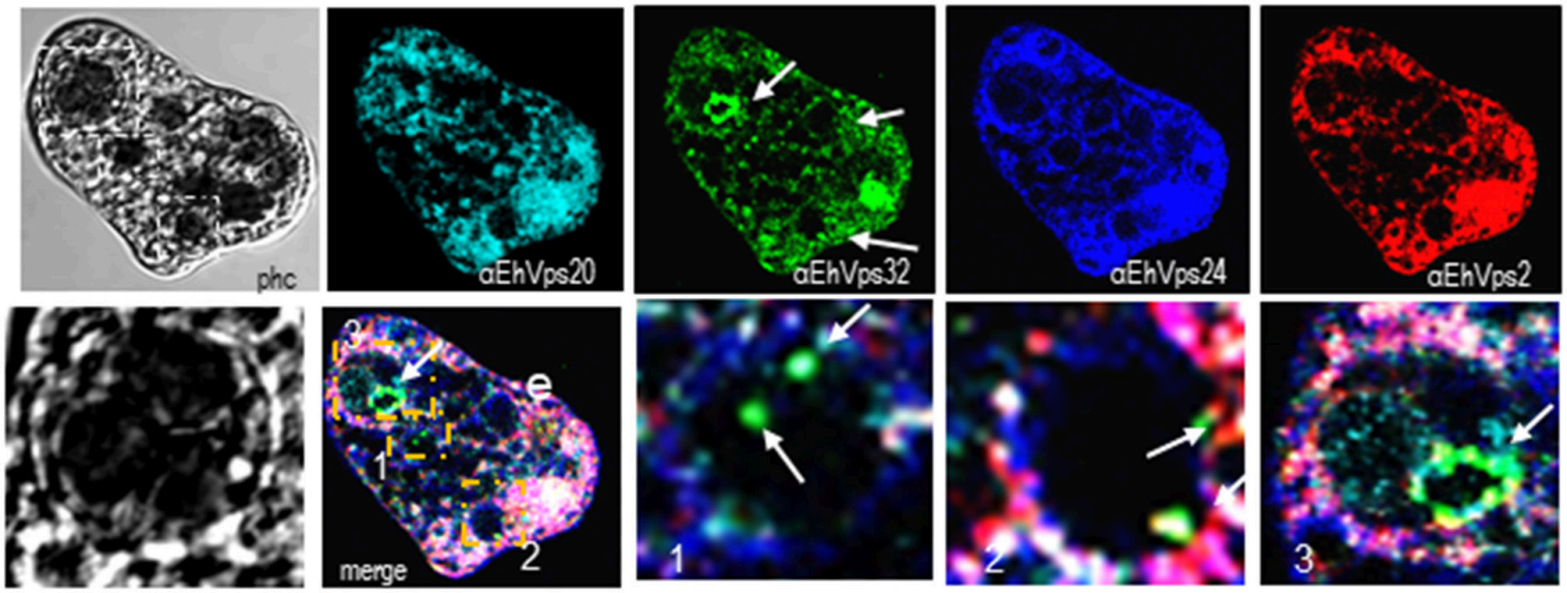
ESCRT-III proteins are present in MVBs like structures of trophozoites. Representative confocal microscopy image of a trophozoite exhibiting MBVs like structures at distinct maturation phases, MVBs like structures are signaled by arrows in the α-rEhVps32 image. Red square of the phase contrast (phc) image is magnified below the picture. The three MVBs like structuresare in white squares in merging images and marked with 1, 2, and 3 numbers that correspond to the three images at right. Arrows in merging images signal the presence of EhVps32 in the putative MVBs structures. e, erythrocyte; phc, phase contrast. Scale bar: 10 μm.

Interestingly, in a single trophozoite, MVBs frequently appeared with distinct morphology, suggesting that these structures were in different maturation stage (Figure 4, red square). The four ESCRT-III proteins also appeared surrounding MVBs-like structures, but they were observed also inside them and in the nascent ILVs. The most abundant protein in these structures was EhVps32 (Figure 4, green). Magnification in Figure 4 illustrates a representative image with at least three MVB-like structures (up to 10 μm of diameter) in a different maturation process (Figure 4, white arrows). Frequently, in these structures, EhVps32 appeared around nascent vesicles (ILVs) inside MVB-like structures. EhVps2, EhVps20 and EhVps24 were also observed inside or surrounding these putative nascent vesicles (Figure 4).

#### The ESCRT-III proteins associate during phagocytosis

**Figure 5:**
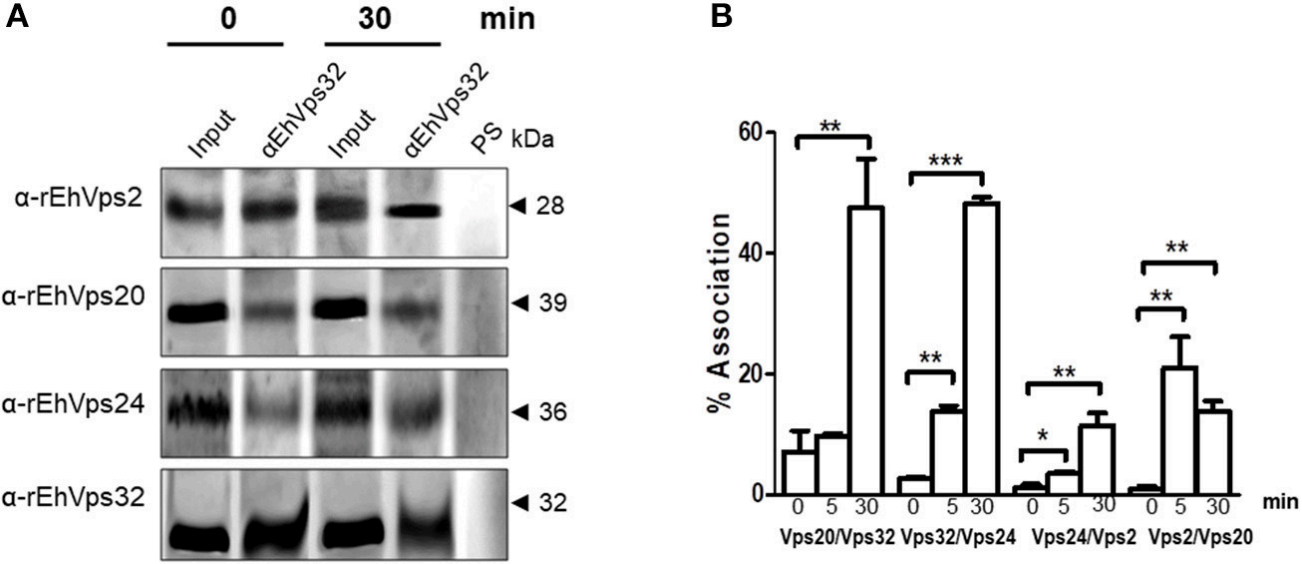
ESCRT-III proteins association increase during phagocytosis. **(A)** Immunoprecipitation using α-rEhVps32 antibodies. Immunoprecipitates were submitted to western blotting assays using antibodies indicated at left. At right: molecular weight of immunodetected proteins. PS: pre-immune serum. Input: trophozoite lysates before immunoprecipitation. **(B)** Association of four couples of ESCRT-III proteins signaled in X-axis in trophozoites in basal conditions and during phagocytosis using the corresponding primary and secondary antibodies. Association was quantified by flow cytometry as described in section Materials and Methods. ^*^p < 0.1, ^**^p < 0.01, ^***^p < 0.001.

We further discern whether the ESCRT-III proteins only co-localized, as confocal images shown, or they associate during phagocytosis. For these experiments we used the α-rEhVps32 antibody to immunoprecipitate lysates from trophozoites in basal conditions and after phagocytosis. Western blot assays of the immunoprecipitates evidenced that the four ESCRT-III proteins associate in basal conditions and during erythrophagocytosis (Figure 5A). However, we did not detect significant differences in the protein amount before and after phagocytosis.

To quantify the protein association, we measured by flow cytometry the interactions between two proteins at the same time, using the sequence described above: (EhVps20/EhVps32, EhVps32/EhVps24, EhVps24/ EhVps2, and EhVps2/EhVps20). Results showed a low percentage of association at basal conditions, slightly higher for EhVps20/EhVps32 (8%) than for the other three couples (2–4%) (Figure 5B). All couples increased their association during phagocytosis. After 30 min of erythrophagocytosis, EhVps20/EhVps32 and EhVps32/EhVps24 reached about 47% interaction, whereas EhVps24/EhVps2 and EhVps2/EhVps20 presented 10 and 13% respectively (Figure 5B). Intriguingly, EhVps2/EhVps20 interaction increased from 2% at basal conditions to 24% at 5 min and decreased to about 12% after 30 min of phagocytosis (Figure 5B). These results suggested that EhVps20/EhVps32 as well as EhVps32/EhVps24 association is stronger than the ones in which EhVps2 is involved. We are aware that protein association is a dynamic process, according to the biological conditions of the trophozoites. Thus, from these experiments, it is not possible to deduce the precise order of protein assembly.

### The EhVps20 (active form) binds more efficiently to artificial membranes than other ESCRT-III proteins

To resolve the sequence in which proteins bind to the membrane and monitor the associated changes in the membrane morphology after their binding, we employed GUVs as a model system (Wollert et al., 2009). The advantage of GUVs over other membrane models is that the membrane response can be directly observed under the microscope. We mimicked the endosomal membrane composition (Evans and Hardison, 1985; Kobayashi et al., 2003) (POPC:POPS:chol:PI(3)P in a 62:10:25:3 ratio) and also tested other lipid mixtures (only DOPC; DOPC:PI(3)P 95:5; DOPC:DOPS 90:10 or DOPC:DOPG 90:10) (for abbreviations see Materials and Methods section); and probed the binding and membrane reshaping produced by *E. histolytica* ESCRT-III recombinant proteins. The GUVs were labeled with the fluorescent lipid analog TexasRed-DHPE to visualize membrane deformations.

Numerous studies have shown that ESCRT-III proteins share a common domain structure of four α-helices packed into an N-terminal core domain and a C-terminal auto-inhibitory region that blocks the main membrane contact sites present in α1 and α2. Truncation of the auto-inhibitory C-terminal region (corresponding to the last α-helix including the k-linker) produces proteins with exposed membrane binding domains (Zamborlini et al., 2006; Shim et al., 2007; Henne et al., 2012).

**Figure 6:**
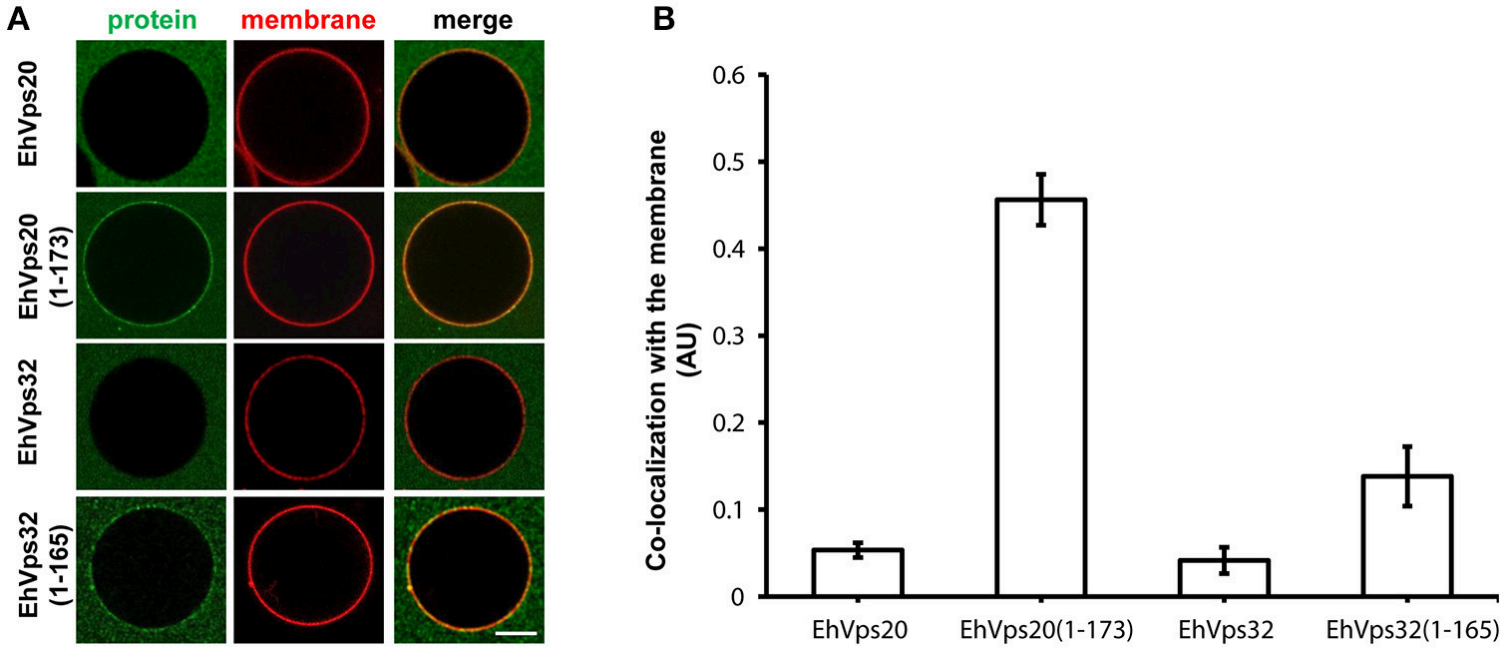
EhVps20 active form binds more efficiently to GUVs than EhVps32. **(A)** GUVs prepared from POPC (62 mol%), POPS (10 mol%), chol (25 mol%), PI(3)P (3 mol%), and TR-DHPE (0.1 mol%) were incubated with 125 nM of each Alexa488 labeled recombinant protein indicated at left for 5 min. GUVs were analyzed through confocal microscope. Scale bar: 10 μm. **(B)** Co-localization of recombinant proteins with Texas-Red labeled membrane in 100 GUVs. Bars indicate average Pearson's coefficient. AU, arbitrary units.

To avoid the use of other factors required for activation, we generated truncated recombinant protein versions of rEhVps20 (1-173 amino acids) and rEhVps32 (1-165 amino acids) (Figure 2A, lanes 3 and 6) to have them in an open conformation. As expected, fluorescently-labeled rEhVps20 and rEhVps32 full proteins presented low binding efficiency to GUVs with poor Pearson coefficients (PC) (Figures 6A,B). In contrast, the active form of fluorescently-labeled rEhVps20 (1-173) exhibited 8.5 times higher binding efficiency to GUVs than the inactive protein (PC = 0.456 and 0.0534, respectively) (Figures 6A,B). The binding efficiency observed with rEhVps32 (1-165) was 3.3 times greater than the triggered by the full protein (PC = 0.1382 and 0.041, respectively) (Figures 6A,B). Interestingly, rEhVps20(1-173) presented 3.26 times greater binding efficiency to GUVs than rEhVps32(1-165) (Figures 6A,B). These experiments showed that only EhVps20(1-173) and EhVps32(1-165) active forms interacted with membranes, but with distinct affinity. Because of the higher binding efficiency exhibited by rEhVps20(1-173), we employed the truncated version of rEhVps20 for the rest of the experiments. It is important to point out that *in vitro* and *in vivo* assays give accuracy to the results obtained, however, results could slightly differ, mainly due to the distinct complexity of the endocytosis/phagocytosis processes and to the differences in membrane composition.

### Reconstruction of the *E. histolytica* ESCRT-III machinery in GUVs

**Figure 7:**
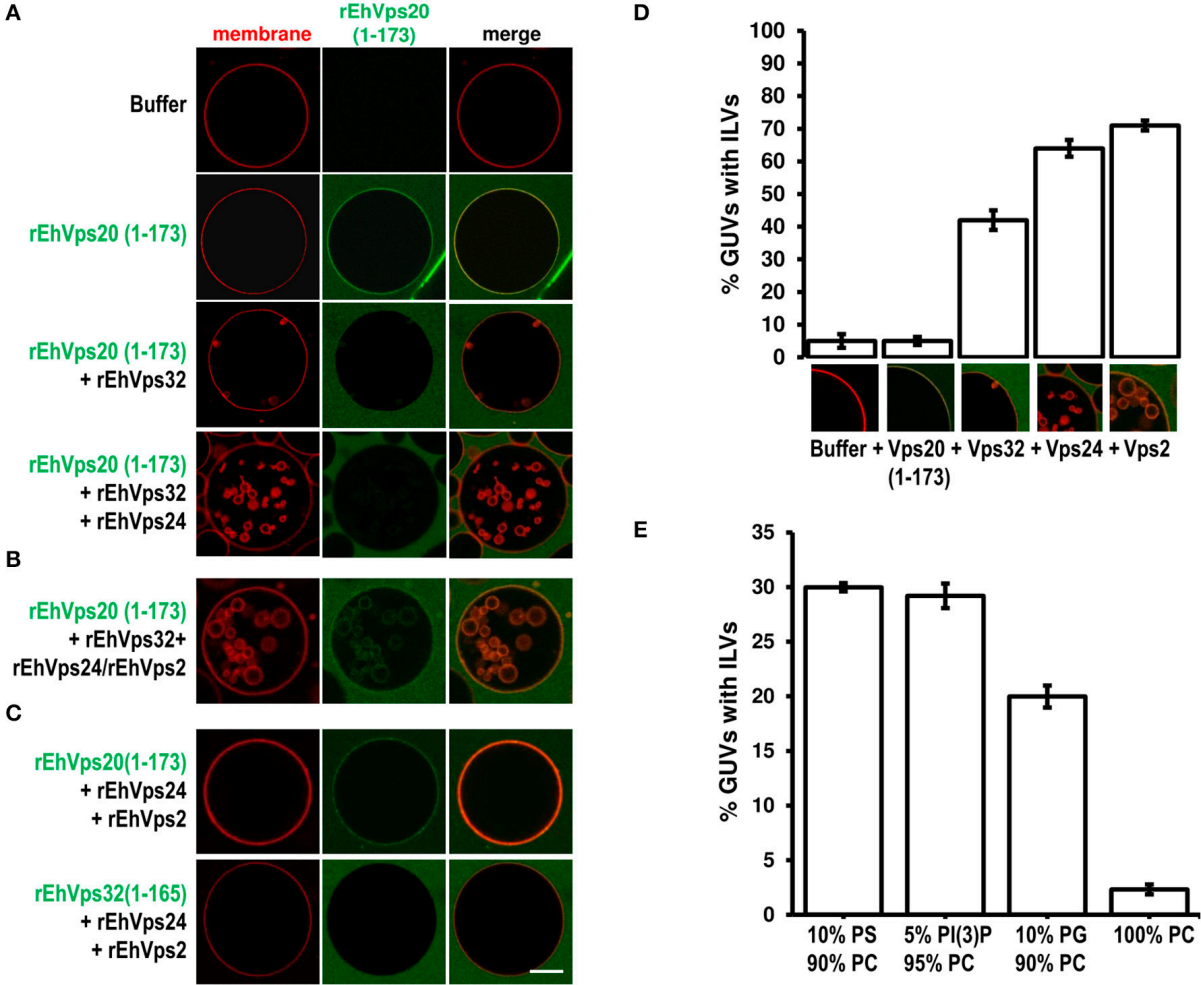
EhVps20, EhVps32 and EhVps24 are required to produce ILVs in GUVs and EhVps2 apparently modulates their size. **(A)** GUVs with the same composition as in Figure 6 and each recombinant protein were added and incubated at RT with 10 min intervals. As a negative control, GUVs were incubated for the same times replacing proteins with buffer. **(B,C)** Experiments where some protein was omitted or substituted as indicated at right. Scale bar: 10 μm. **(D)** Quantification of the number of GUVs containing ILVs formed for subsequent addition of the proteins indicated below. In the bottom of each bar images of ILVs formation after each protein addition are shown. **(E)** GUVs prepared with DOPC alone or a mixture of different negatively charged lipids (negative molar ratio of 10%) were incubated with the four ESCRT-III proteins. Bars represent the mean and standard error of three independent experiments.

To rebuild the whole ESCRT-III machinery and establish the binding order of the proteins, we added in distinct sequence each of the ESCRT-III recombinant proteins to TexasRed-DHPE-labeled GUVs, giving 5 min intervals between the additions of each protein. As previously observed, confocal images showed that Alexa488 labeled rEhVps20(1-173) binds to GUV membranes (Figure 7A). After adding rEhVps32, we detected small fluorescent quasi-spherical buds attached to the rEhVps20-labeled membranes, suggesting invaginations induced by rEhVps32. After 10 min, the buds remained attached to GUV membranes via narrow membrane necks, but when we incorporated the rEhVps24 protein, small ILVs (~0.5 μm) were generated inside the GUVs (Figures 7A,B). It is quite remarkable that all quasi-spherical buds induced by the binding of rEhVps20 and rEhVps32 were similar in size. Such a uniform bud size indicates that the adsorbed protein layer undergoes phase separation into a rEhVps32-rich and a rEhVps32-poor phase and that the rEhVps32-rich domains have a significant spontaneous curvature that determines the bud size (Lipowsky, 1992).

The results presented so far could imply that EhVps2 is not necessary for ILVs generation. However, upon incubation of GUVs with EhVps20(1-173) and EhVps32 followed by the addition of rEhVps24 and rEhVps2 together, many larger ILVs appeared (~3 μm of diameter) (Figure 7B). Additionally, the percentage of GUVs with ILVs increased from 64 to 71% by EhVps2 presence (Figure 7D). These results strongly suggest that in *E. histolytica*, the core formed by active EhVps20, EhVps32, and EhVps24 is sufficient to generate ILVs, while EhVps2 acts as an additional regulator of ILVs size. The substitution of EhVps20(1-173) by EhVps32(1-165) did not produce ILVs, revealing that the function of EhVps20 and EhVps32 is not redundant (Figure 7C). Experiments changing the sequence of the proteins (Figure S1) indicated that the order of protein addition to produce ILVs must be the following: active EhVps20, EhVps32, EhVps24, and EhVps2.

To prove the specificity of ESCRT-III binding toward negatively charged membranes, we performed reconstruction assays using GUVs entirely composed of DOPC (zwitterionic) or a combination of the same negative molar ratio (equivalent to 10% PS) of different negatively charged lipids: DOPS, PI(3)P and DOPG. In eukaryotes, PS and PI(3)P are present on early endosomes and the internal leaflet of the plasma membrane (Gillooly et al., 2000) while PG is present in non-endosomal-related membranes such as mitochondrial. As expected, ILVs appeared only in negatively charged membranes (Figure 7E). We also found that rEhVps20(1-173) formed protein clusters on the surface of membranes with high negative charge (data not shown). The greatest percentage of ILV formation was achieved using GUVs with DOPS and PI(3)P (~30% in both cases) (Figure 7E). These results suggested that even when ESCRT-III proteins are able to bind to a wide range of different negatively-charged lipids, there is an apparent affinity toward lipids present in endosomal membranes during the transition from early to late endosomes (Lemmon and Traub, 2000).

### Knock down of *Ehvps20* and *Ehvps24* genes affects the rate of phagocytosis in trophozoites

**Figure 8:**
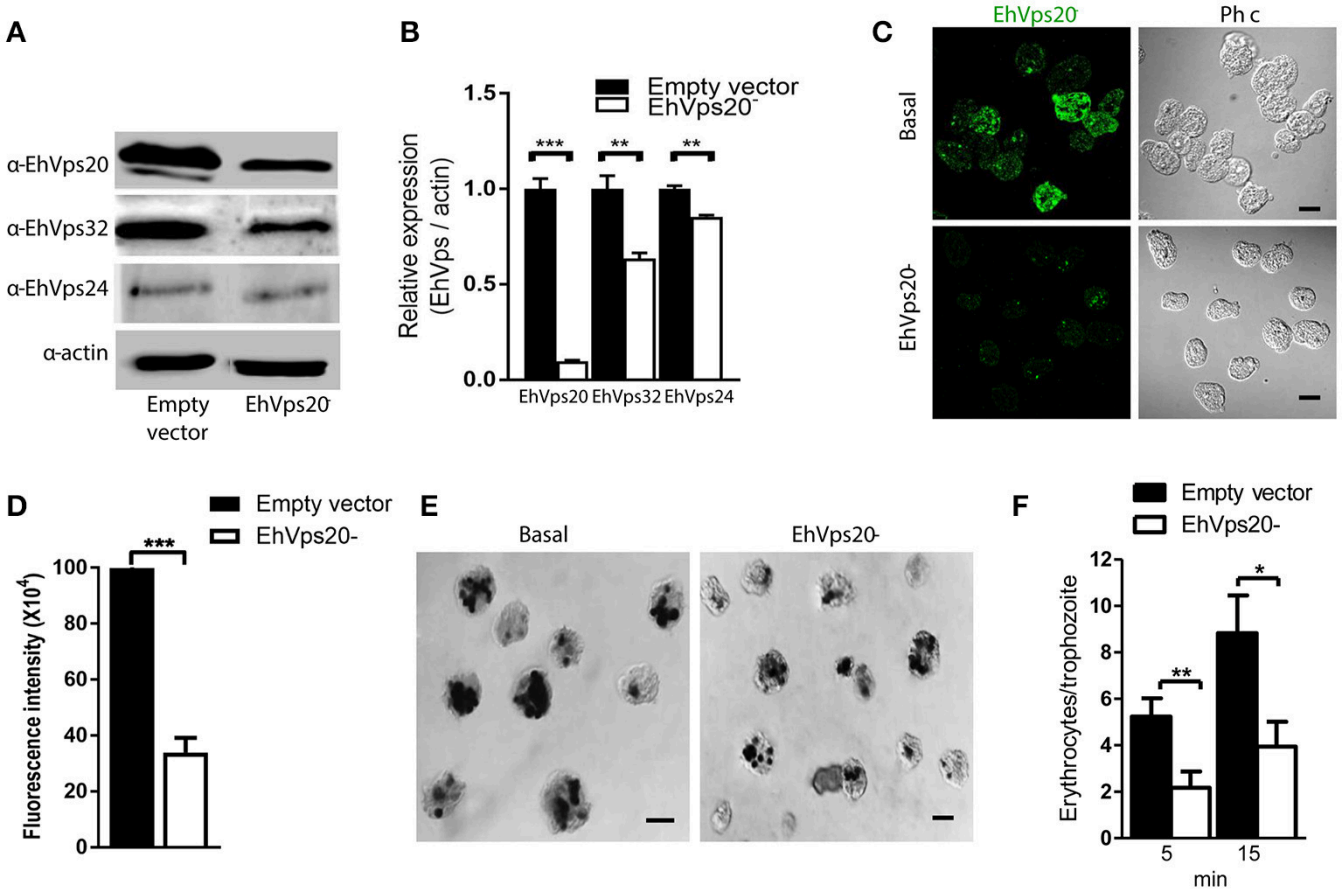
Knock down of *EhVps20* gene affects the rate of erythrophagocytosis. Trophozoites of G3 clone were transfected with *pSAP2/GunmaEhVps20* plasmid (*EhVps20*^−^) or with the empty vector (vector). **(A)** Western blot of transfected trophozoites lysates, using antibodies indicated at left. As a loading control, the same membrane was re-blotted with α-actin antibodies. **(B)** Densitometry analysis of bands showed in **(A)**. **(C)** Representative image of confocal microscopy and phase contrast of transfected trophozoites using α-rEhVps20 antibody and TRITC-labeled secondary antibody. **(D)** Fluorescence intensity measured by pixels. **(E)** Novikoff stained trophozoites that ingested erythrocytes for 15 min. Scale bar: 10 μm. **(F)** Rate of erythrophagocytosis of transfected trophozoites. Data represent the mean and standard error of the erythrocytes number counted inside of 100 trophozoites. ^*^p < 0.05, ^**^p < 0.01, ^***^p < 0.001.

To get more evidence on the role of ESCRT-III complex in phagocytosis of trophozoites, first, we knocked down the *Ehvps20* gene, whose product is the first protein that binds to membranes and triggers the assembly of the rest of ESCRT-III members. We employed trophozoites of the G3 strain to transcriptionally silence the *Ehvps20* gene (Mirelman et al., 2006). Western blot assays revealed 60% protein reduction in *EhVps20*^−^silenced trophozoites compared with those transfected with the empty vector (Figures 8A,B). The expression of other ESCRT-III members, was significantly affected (Figure 8B). Immunofluorescence assays confirmed the reduction of the EhVps20 amount in *Ehvps20*^−^ trophozoites (Figures 8C,D). These trophozoites, exhibited a reduced capacity to ingest erythrocytes (65–70% at 5 min and 50–65% at 15 min), compared with the rate of ingestion of G3 trophozoites transfected with the empty vector (Figures 8E,F).

**Figure 9:**
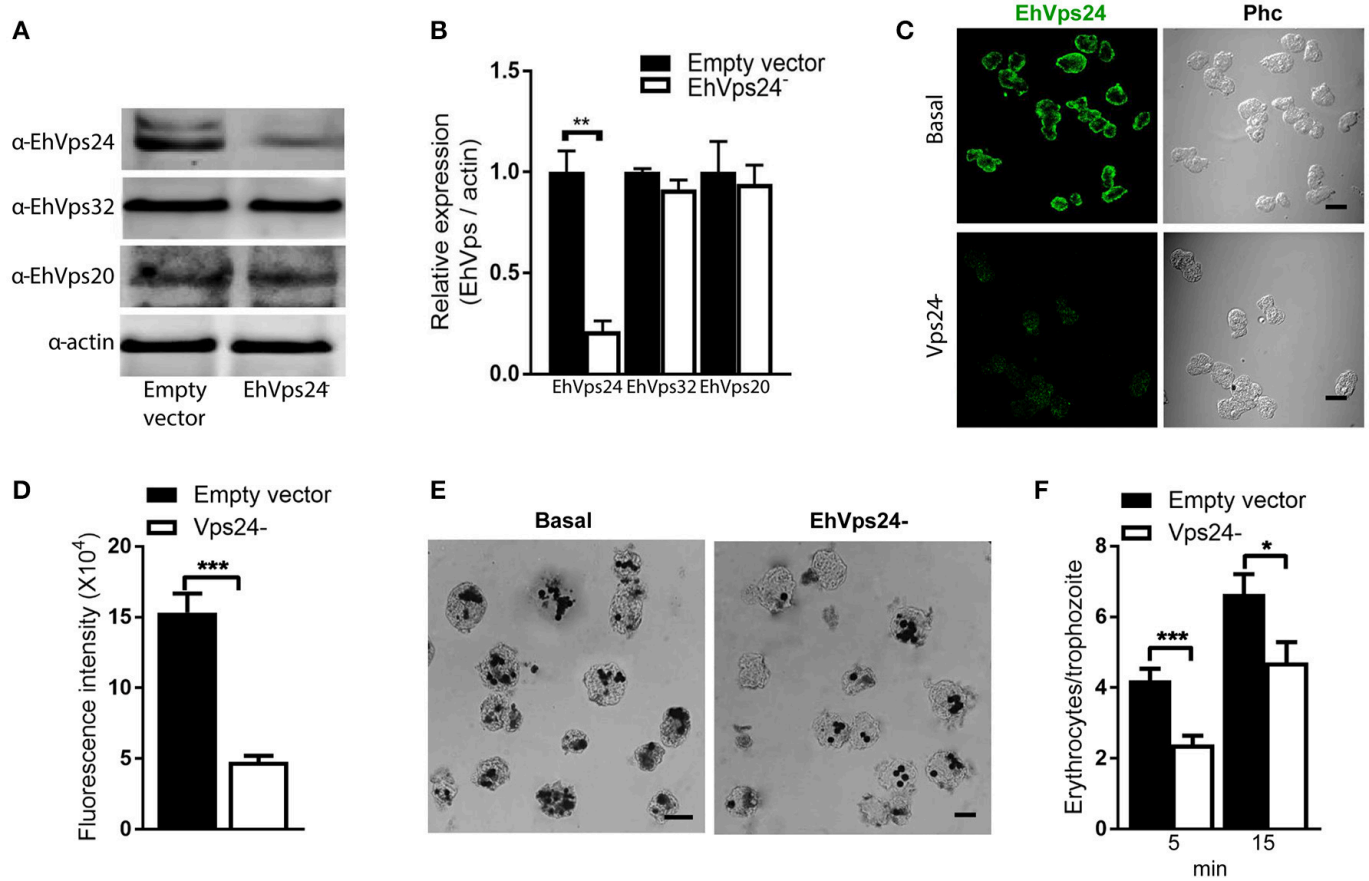
Knock down of *EhVps24*gene affects the rate of erythrophagocytosis. Trophozoites of G3 clone were transfected with *pSAP2/GunmaEhVps24* plasmid (*EhVps24*^−^) or with the empty vector (vector). **(A)** Western blot of transfected trophozoites lysates, using antibodies indicated at left. As a loading control, the same membrane was re-blotted with α-actin antibodies. **(B)** Densitometry analysis of bands showed in **(A)**. **(C)** Representative image of confocal microscopy and phase contrast of transfected trophozoites using α-rEhVps24 antibody and TRITC-labeled secondary antibody. **(D)** Fluorescence intensity measured by pixels. **(E)** Novikoff stained trophozoites that ingested erythrocytes for 15 min. Scale bar: 10 μm. **(F)** Rate of erythrophagocytosis of transfected trophozoites. Data represent the mean and standard error of the erythrocytes number counted inside of 100 trophozoites. ^*^p < 0.05, ^**^p < 0.01, ^***^p < 0.001.

The knocked down of *Ehvps24* gene showed 25% remnant expression of the protein, using as control the actin protein (Figures 9A,B). Silencing of this gene did not affect the expression of the EhVps32 and the EhVps24 proteins (Figures 9A,B). Immunofluorescence assays confirmed the 75% reduction of fluorescence in *Ehvps24*^−^ knocked down trophozoites, in comparison with trophozoites transfected only with the vector (Figures 9C,D). On the other hand, the rate of erythrophagocytosis of *Ehvps24*^−^knocked down trophozoites appeared affected in about 60% at 5 min and 66% at 15 min (Figures 9E,F). These results, together with the ones previously obtained with *EhVps32* knocked down trophozoites (Avalos-Padilla et al., 2015) strengthened the hypothesis that ESCRT-III in *E. histolytica* has prior influence in the rate of phagocytosis in the trophozoites. Each one of its member carry out defined functions that are necessary to efficiently ingest and process target cells.

## Discussion

The ancient ESCRT machinery is critical for several central cellular processes; among them, endocytosis, MVBs biogenesis, viral budding, cytokinesis, autophagy, exosomes secretion and others based on membrane deformation and scission (Carlton and Martin-Serrano, 2007; Filimonenko et al., 2007; Lee et al., 2007; Rusten et al., 2007). However, its role in phagocytosis, an event in which membrane deformation and scission is evident, has not been completely explored. Besides, many of the detailed molecular mechanisms to produce these events are unknown. But it is well documented that process involving membrane remodeling are linked to the assembly-disassembly cycles of ESCRT-III proteins in membranes (Hurley, 2015). Thus, it is expected that ESCRT machinery has a relevant role in the phagocytosis of the *E. histolytica* trophozoites.

In *E. histolytica*, the precise function for the majority of the ESCRT products is unknown, even when almost all ESCRT genes are present in the genome (López-Reyes et al., 2011). EhVps32, the ortholog of Snf7 in yeast and CHMP4 in human, as well as the accessory proteins EhADH (an ALIX family protein) and the EhVps4 ATPase are involved in the phagocytosis of trophozoites (García-Rivera et al., 1999; López-Reyes et al., 2010; Avalos-Padilla et al., 2015). Additionally, EhVps32 and EhADH are also involved in pinocytosis of dextran and phagocytosis of latex-coated beads (Avalos-Padilla et al., 2015; Castellanos-Castro et al., 2016) which implies that ESCRT-III proteins participate in different types of endocytosis. Even when substantial work has been done in the study of the molecules involved in phagocytosis in *E. histolytica* (Petri et al., 2002; Saito-Nakano et al., 2004; Seigneur et al., 2005; Jain et al., 2008), many others, as well as the mechanisms participating from the interaction with cargo molecules to the digestion and recycling of proteins, are not well known yet. Here, we expanded our research on *E. histolytica* phagocytosis, using *in silico* analysis, mutant trophozoites, ESCRT-III recombinant proteins and the GUV model to unravel a part of the phagocytosis puzzle. Our results give evidence that the ESCRT-III proteins participate in the formation of phagocytic cups, phagosome maturation and MVB-like structure generation in this primeval voracious parasite.

*In silico* analysis showed that *E. histolytica* possesses all four ESCRT-III proteins with a 3D structure similar to the yeast and human orthologs. Although their amino acid sequences exhibit low homology (less than 32% in all cases, Table 1), the four proteins have all functional domains described in their orthologs (Figures 1A,B), remarking the importance of these domains in distinct cellular events and their conserved function in eukaryotes. The high homology in the secondary protein structure together with their function performed during phagocytosis, reconstructed in GUVs, confirmed that they are *bona fide E. histolytica* orthologs of ESCRT-III proteins.

Immunofluorescence assays, using specific antibodies against each of the four ESCRT-III proteins evidenced that all they make contact with the erythrocytes since early times of erythrophagocytosis (Figures 2C, 3B). Even in basal conditions, images revealed proteins co-localizing in all possible combinations, due to the active constitutive endocytosis of trophozoites (Figure 3A). Moreover, immunoprecipitation and flow cytometry experiments using α-rEhVps32 antibody revealed that, in basal conditions and during phagocytosis, the proteins associate with each other and association is enhanced during phagocytosis (Figure 5). Although we cannot discern whether it is a direct or indirect association, results strongly support their involvement in this event and evidence the dynamic of the protein association.

**Figure 10:**
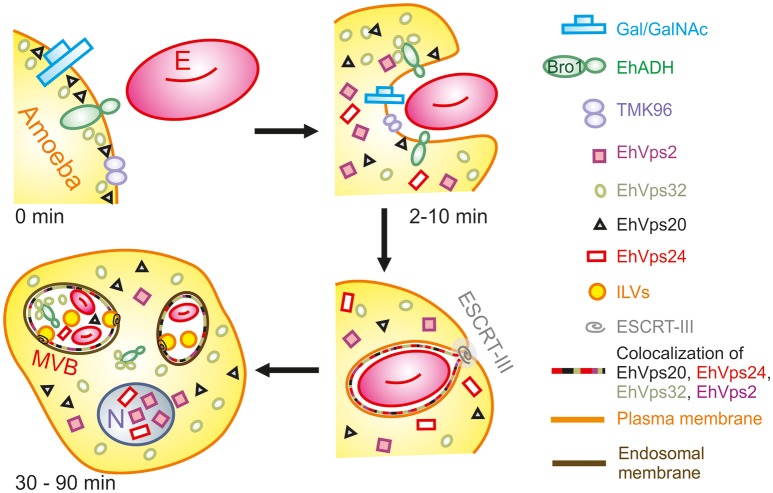
Model for the participation of ESCRT-III in phagocytosis. (0 min) Attachment to human erythrocytes to trophozoites involves plasma membrane proteins including Gal/GalNAc lectin, TMK96 and EhADH. In particular, EhADH possesses a Bro1 domain in the cytoplasmic tail that recruits EhVps32 upon binding of erythrocytes to trophozoites. In this first phase of contact, it is also possible to observe EhVps20 in close contact with the plasma membrane. (2–10 min) The union of erythrocytes triggers signaling mechanisms that modulate actin cytoskeleton that together with other proteins remodels and produces the phagocytic cup. During this stage, EhADH-EhVps32 complex and EhVps20 independently recruit the other ESCRT-III molecules to the phagocytic cup and surround the nascent phagosome forming hetero-polymers that will finally lead to the scission and internalization of the phagosome. (30–90 min) After longer times, huge phagosomes with different number of erythrocytes inside them appear as a product of the fusion of several phagosomes. In such phagosomes, the formation of ILVs is triggered by the action of ESCRT-III polymers and the proteins are present either surrounding the phagosome, or inside them in close contact with the internalized erythrocytes. After 60 min of phagocytosis, EhVps2 and EhVps24 consistently migrated to the nucleus. E, erythrocyte.

During the early stages of phagocytosis (10 min), EhVps32 and EhVps2 consistently appeared in the phagocytic cups and in the plasma membrane close to the contact with the erythrocytes (Figure 10). This location suggests a novel function for these two proteins, not described in other eukaryotes, during the capture of the cargo, in addition to their known role in the ESCRT-III-complex formation on endosomes. At the first contact of trophozoites with erythrocytes, EhVps32 co-localize with Gal/Gal lectin and EhADH, which can act as receptors for erythrocytes in the early phagosome maturation stages (Avalos-Padilla et al., 2015) (Figure 10). Thus, the assembling of the phagocytosis puzzle in *E. histolytica* has advanced with the results shown here.

Later in phagocytosis, when trophozoites have ingested a larger number of erythrocytes, EhVps20 covered the erythrocytes inside phagosomes, and EhVps32 together with EhVps24 surrounded them. EhVps2 also appeared around the phagosomes (Figure 4). Intriguingly, EhVps24 and EhVps2 consistently migrate to the nucleus during phagocytosis, when digestion of the prey is advanced (Figure 2D). The nuclear localization of these proteins was confirmed by co-localization with the nuclear protein pCNA (Figure 2E) So far, we have no experimental data to speculate about the nuclear function of these proteins, although ESCRT-III proteins participate in nuclear envelope repair in human cells (Raab et al., 2016; Ventimiglia and Martin-Serrano, 2016). As migration happens in more than 95% of the trophozoites, we conjectured that they could act as transcription factors or co-factors or to repair the nuclear envelope as in humans. Moreover, phagocytosis is an exhausting experience for trophozoites; it produces stress, and involves and consumes many cellular proteins. EhVps24 and EhVps2 could contribute by a yet unknown mechanism to recover the basal state of trophozoites after long times of phagocytosis. This could involve transcripts synthesis to replace the proteins consumed during the process. However, further experiments will help to better understand this.

The ESCRT-III proteins in trophozoites in basal conditions and during phagocytosis showed a highly dynamic movement in the cell, accordingly to the synthesis and exchange of membranes in this insatiable parasite. Confocal images revealed the detection of ESCRT-III proteins in distinct forms of phagosomes and MVBs-like structures, which might correspond to different phases of phagosome maturation (Figure 4), as it is shown in Figure 10. In other systems, ESCRT-III proteins start ILVs formation at an intermediate stage, where the phagosome is no longer an early compartment but has not yet the characteristic features of a late phagosome (Flannagan et al., 2012). It is possible to hypothesize that this also occurs in trophozoites. At 60 and 90 min, when digestion is advanced, we detected ESCRT-III proteins in MVB-like structures (up to 10 μm of diameter) with partially digested erythrocytes and a distinct number of ILVs and phagosomes with different shapes. In other eukaryotes, MVBs are smaller (400–500 nm of diameter); however, they are mainly formed during receptor-mediated endocytosis of molecules and small particles, whereas the erythrocytes are much larger than the ingested molecules during these types of endocytosis. Besides, phagosomes can content between one to twenty or more erythrocytes. Interestingly, in these MVBs-like structures, we consistently observed EhVps32 clusters in the membrane (Figure 4), which might correspond to the nascent vesicles observed in GUVs after contact with active rEhVps20 and rEhVps32 (Figure 7). We also detected the presence of EhVps24 and EhVps2 in these invaginations suggesting that the four proteins participate in ILVs formation in trophozoites.

Phagocytosis is a process that occurs in a series of chained events and a large number of proteins participate in it. This makes difficult to assign by *in vivo* experiments, a particular function to each protein. The GUV model and the recombinant proteins allowed us to precisely assign a function for each one of the *E. histolytica* ESCRT-III proteins during ILVs formation and to dissect the order in which proteins are assembled. We are aware that, as in other systems, ESCRT-III proteins could perform other functions, unidentified here. The fact that the recombinant proteins bind differentially to GUV membranes shows the adequacy of the model for this type of studies.

By the GUV model we confirmed that active rEhVps20(1-173) efficiently binds only to negatively charged membranes, and exhibit no specific preference toward the charged lipid species which can explain its presence in different membranes. Then, rEhVps32 binds to rEhVps20 and produces quasi-spherical membrane buds connected to the GUVs by narrow membrane necks. The uniform size of these buds indicates that the adsorbed protein layer undergoes phase separation and forms rEhVps32-rich domains with a significant spontaneous curvature that determines the bud size (Lipowsky, 1992). The two proteins and the sequence of their binding are necessary to initiate ILV generation, but rEhVps20 and rEhVps32 do not cleave the neck to release ILVs. As in their orthologs, EhVps32 and EhVps24 are able to polymerize, and polymers could nucleate in the areas where the membrane is deformed. Then, the polymer growth can provoke the closure of the formed neck and split the nascent vesicle producing ILVs.

Our experiments also evidenced that EhVps24 in combination with rEhVps2 modulate the size of the ILVs. It is interesting that EhVps2 appears to increase the size of the generated ILV, this function is especially important in the case of phagocytosis, where the diameter of ILVs within phagosomes should be larger due to the proportion of the ingested cells. We do not know to what extent EhVps2 modulates size and EhVps24 cleaves the nascent vesicles in trophozoites, but we can speculate that they have analogous functions as revealed in GUV experiments.

Finally, the knock down of *Ehvps20* and *Ehvps24*, presented in this paper, and *Ehvps32*, published earlier (Avalos-Padilla et al., 2015), genes in trophozoites confirmed the functional importance of the ESCRT-III complex in phagocytosis, since EhVps20 is necessarily the first protein that binds to membranes to recruits the others. Its silencing reduced the rate of phagocytosis in transfected trophozoites (Figure 8), evidencing its importance in this step of the phagocytosis. Interestingly, the knock-down of the *Ehvps20* gene, affected the expression of the EhVps32 and EhVps24 proteins. Since EhVps20 is the first protein that recruits the rest of the ESCRT-III subunits, its absence can affect the expression of the rest of the ESCRT-III members. In the same way, the knocked down of *EhVps24*, an important player in ILVs formation, affected the rate of phagocytosis, but had no effect in the expression of the other ESCRT-III members. The remnant phagocytic activity in transfected trophozoites can be due to the action of the remnant protein and to other proteins involved in phagocytosis, among them the Rab family proteins, which act as regulators or carriers of other molecules (Rodríguez and Orozco, 2000; Saito-Nakano et al., 2004). Moreover, in previous experiments, the silencing of EhVps32 impaired the rate of phagocytosis in 80% in trophozoites (Avalos-Padilla et al., 2015), these differences could be due to the distinct participation of the proteins as seen in the *in vitro* studies in this paper. Considering that EhVps32 also act during capture of cargo and together with EhADH (Avalos-Padilla et al., 2015), it can participate in an alternative pathway of ESCRT-III proteins recruitment. Since phagocytosis is a complex process, the GUVs model is suitable to analyze the specific function of each of the proteins that participate in phagocytosis/endocytosis and their relationship to other molecules already studied.

In conclusion, we have been able to reconstruct the sequential assembling of ESCRT-III and give evidences for its participation in the phagocytosis of *E. histolytica*, as it is shown in the model in Figure 10. Additionally, we demonstrate here that the combination of *in vivo* and *in vitro* studies using GUVs is a good strategy to analyze with high detail the role of these proteins in membrane deforming. Also this is a good model to study virulence functions in parasites.

## Materials and methods

### *E. histolytica* cultures

Trophozoites of *E. histolytica* (strain HM1:IMSS) clones A (Orozco et al., 1983) and G3 (Mirelman et al., 2008) were axenically cultured in TYI-S-33 medium at 37°C and harvested in logarithmic growth phase (Diamond et al., 1978) to perform the experiments. *S*ilenced G3 trophozoites were analyzed 24 h after transfection (Mirelman et al., 2008). Experiments were performed at least three times by duplicate for statistical analysis.

### *In silico* analysis of EhVps20

The SNF7 protein domain was searched in the *E. histolytica* genome database (www.amoebadb.org). Comparison between putative EhVps20 protein and human and yeast orthologs was determined using the Expert Protein Analysis System (ExPASy) Proteomics Server by the NCBI BLAST service program. Sequence alignments were generated using the ClustalW2 program (Blackshields et al., 2006).

### Tertiary (3D) protein modeling

EhVps2, EhVps20, and EhVps24 amino acid sequences were submitted to the Phyre2 Server (http://www.sbg.bio.ic.ac.uk/phyre2/html/page.cgi?id=index) to obtain the proteins 3D predicted structures. Results obtained were documented and analyzed through the UCSF Chimera software (Pettersen et al., 2004).

### Cloning and expression of recombinant proteins

The full-length *Ehvps2, Ehvps20 and Ehvps24* genes, the first 173 residues from *Ehvps20* and the first 165 residues from *Ehvps32* were PCR-amplified using cDNA as template and specific primers (Table 1) which introduced unique *BamHI* and *SalI* sites in the sense and antisense primers, respectively (underlined in Table 2). Genes were cloned into the *pJET1.2/blunt* plasmid (Thermo Fisher, Waltham, MA, USA), accordingly to manufacturer's instructions. Then, genes were subcloned into the *BamHI* and *SalI* sites of the *pGEX-6P-1* plasmid, generating *pGEX6P-Ehvps2, pGEX6P-Ehvps20, pGEX6P-Ehvps20(1-173), pGEX6P-Ehvps24* and *pGEX6P-Ehvps32(1-165)* constructs. *Escherichia coli pLys-S* bacteria were transformed with the plasmids, and recombinant proteins were induced by 0.1 mM of IPTG to produce GST-rEhVps2, GST-rEhVps20, GST-rEhVps20(1-173), GST-rEhVps24 and GST-rEhVps32(1-165) tagged proteins. GST-tagged proteins were dialyzed against the buffer for the PreScission protease enzyme (GE-healthcare, Freiburg, Germany) and the GST-tags were removed according to manufacturer's instructions. GST-free proteins were purified by size exclusion chromatography.

**Table 2:** Primers used for genes amplification.

**Gene**	**size**	**Sense primer**	**Antisense primer**
*Ehvps2*	741	5′-GCGGATCCATGTCAAGGTTATTC-3′	5′-CCGTCGACTTAAAGATTGGCTATTCT
			TGAAACAATATCATCTTCATCTTC-3′
*Ehvps20*	621	5′-GCGGATCCATGTTAAATCGATTCA-3′	5′-CCGTCGACTTTAAATTGCAAATTTTTT
			GCTATCTGGTT-3′
*Ehvps20 (1-173)*	522	5′-CCGGATCCATGTTAAATCGATTCA-3′	5′-CGTCGACTTAAATATCTCCTTCAAACA
			TTGAAT-3′
*Ehvps24*	618	5′-GCGGATCCATGGGCAACCTTAAT	5′-CCGTCGACTTAAAAGGTTTCTATTACA
		AGCCAAACAGTAGATAATAG-3′	ATATTTTGTTTAGATATTAACATTCACTT-3′
*Ehvps32 (1-165)*	495	5′-CCCCGGATCCATGTCTTGGTTCA	5′-CCCCGTCGACTTATTCTTGTTCATCA
		GAAGAAATACTAC-3′	AGAACTTGATCTTCTAAT-3′

### Generation of polyclonal antibodies

rEhVps2 (60 μg) emulsified in Titer-Max Classic adjuvant (1:1) (Sigma, St. Louis, MO, USA) was subcutaneously and intramuscularly inoculated into *Wistar* rats. Two more doses of rEhVps2 (30 μg) were injected at 20 days intervals and then, animals were bled to obtain α-rEhVps2 antibodies. rEhVps20 and rEhVps24 proteins were immunized in *New Zealand* male rabbits following the protocol previously described using 100 μg of protein for the first dose and 50 μg for subsequent doses. α-rEhVps32 was previously generated in mice (Avalos-Padilla et al., 2015). Pre-immune serum was obtained before immunization in all cases.

### Western blot experiments

Trophozoites lysates (30 μg) were separated in 12% sodium dodecyl sulfate polyacrylamide gel electrophoresis (SDS-PAGE), transferred to nitrocellulose membranes and probed with mouse α-rEhVps32 (1:15,000) (Avalos-Padilla et al., 2015), rat α-rEhVps2 (1:15,000), rabbit α-rEhVps20 (1:20,000) or rabbit α-rEhVps24 (1:10,000) antibodies. Membranes were washed, incubated with the corresponding α-mouse, α-rat or α-rabbit HRP-labeled secondary antibodies (Zymed; 1:10,000), and revealed with ECL Prime western blotting detection reagent (GE-Healthcare).

### Laser confocal microscopy assays

Trophozoites were grown on coverslips, fixed with 4% paraformaldehyde (PFA) at 37°C for 1h, permeabilized with 0.2% Triton X-100 and blocked with 10% fetal bovine serum (FBS) in PBS. Then, cells were incubated with either mouse α-rEhVps32 (1:200), rat α-rEhVps2 (1:200), rabbit α-rEhVps20 (1:200) or rabbit α-rEhVps24 (1:200) antibodies at 37°C for 1h, followed by incubation for 1h with α-rat TRITC-labeled, α-mouse or α-rabbit FITC-labeled secondary antibodies (Zymed-Thermo Fisher; 1:100) as appropriate. For multi-labeling experiments, secondary antibodies were used to detect EhVps32 and EhVps2 as above. For rEhVps20 and rEhVps24 detection, no secondary antibodies were used, instead, the α-rEhVps20 antibody was labeled with Alexa647 fluorochrome and the α-rEhVps24 antibody was labeled with AlexaPacific blue kit (Molecular Probes-Thermo Fisher), accordingly to the manufacturer's instructions. In the case of nuclear co-localization, rEhVps2 and EhVps24 were detected by secondary antibodies as described previously. pCNA (Trasviña-Arenas et al., 2017) protein was detected incubating cells with α-pCNA (Trasviña-Arenas et al., 2017) (1:200) for 1h at 37°C, followed by incubation with α-mouse TRITC-labeled antibodies (1:100) at 37°C for 1h. Nuclei were counterstained by 2.5 μg/ml 4′,6-diamidino-2-phenylindole (DAPI; Sigma) for 5 min. All preparations were preserved using Vectashield antifade reagent (Vector, Burlingame, CA, USA), examined through a Carl Zeiss LMS 700 confocal microscope in laser sections of 0.5 μm and processed with ZEN 2009 Light Edition Software (Zeiss, San Diego, CA, USA).

### Phagocytosis assays

Trophozoites were incubated at 37°C with human erythrocytes (1:25 ratio) for different times at 37°C and processed them for immunofluorescence (10, 15, 30, 60, and 90 min), immunoprecipitation (0 and 30 min) and flow cytometry (0, 5, and 30 min). For immunofluorescence experiments, non-ingested erythrocytes were removing using a mixture of TY1-S-33 medium and water (1:1) at 37°C. This methodology permits to distinguish phagocytic cups from pseudopodia and other membrane arrangements, without producing significant damage to the adhered erythrocytes and membrane structures. After 4% PFA fixation, an aliquot of the cell mixture was put on coverslips and processed as described above. For some experiments, to distinguish erythrocytes, preparations were stained by Novikoff technique (Novikoff et al., 1972) and then, we counted the number of ingested erythrocytes per trophozoite in 100 trophozoites. Simultaneously, we measured the amount of hemoglobin inside trophozoites by spectrophotometry at 400 nm as described (Vacca et al., 1978).

### Immunoprecipitation experiments

Trophozoites were lysed with 10 mM Tris-HCl, 50 mM NaCl and 100 mM protease inhibitors (PHMB, IA, NEM and TLCK), followed by cycles of freeze-thawing in liquid nitrogen and vortexing. In parallel, 200 μl of recombinant protein G-agarose (rProtein-G; Invitrogen) were incubated with 100 μg of mouse α-rEhVps32 antibody or pre-immune serum for 2 h at 4°C, with gentle stirring. Then, rProtein-G beads were washed with 0.5% BSA in PBS, followed by additional washes with PBS for 5 min, under gentle stirring and centrifuged at 11,600 × g for 2 min. Trophozoites lysates (1 mg) were pre-cleared with 200 μl of rProtein-G (previously blocked with 2% BSA) and incubated 2 h at 4°C under gentle stirring. Samples were centrifuged at 11,600 × g to obtain the supernatant that was added to rProtein-G previously incubated with the antibody. Preparations were incubated overnight (ON) at 4°C and then, beads were recovered by centrifugation. After washes with PBS, 60 μl of 4X sample buffer (40% glycerol, 240 mM Tris-HCl pH 6.8, 8% SDS, 0.04% bromophenol blue and 5% β-mercaptoethanol) were added. Samples were boiled for 3 min and centrifuged again at 11,600 × g for 2 min at 4°C. Supernatant (30 μl) was loaded into 12% SDS–PAGE and subjected to western blot assays.

### Flow cytometry assays

Trophozoites (2 × 10^6^) in 2 ml of TYI medium at 37°Cwere incubated with erythrocytes for 0, 5, and 30 min as described above. At the end of each time, cell mixtures were centrifuged at 50 × g for 7 min and then, washed with 10 ml of PBS three times. Pellets were PFA (4%) fixed for 1 h and washed again as above. One ml of 0.2% TritonX-100 was added to each pellet for 10 min and subsequently, cells were washed again with PBS and incubated with 10% fetal bovine serum at 37°C for 1 h. After this time, pellets were washed and four couples of the primary antibodies (1:100): α-rEhVps20/α-rEhVps32, α-rEhVps32/α-rEhVps24, α-rEhVps24/α-rEhVps2 or α-rEhVps2/α-rEhVps20 were added separately to the cell mixtures and incubated ON at 4°C. Cells were washed again three times with PBS and incubated for 1 h at 37°C with the corresponding secondary antibodies (1:200) coupled to distinct fluorochromes as follows: α-rEhVps20and α-rEhVps24 with Cy5-labeled α-rabbit (cyan), α-rEhVps32 with Alexa488 α-mouse (green), and α-rEhVps2 with Alexa647 α-rat (red). Cell mixtures were washed three times with PBS, re-suspended in 500 μl of PBS and analyzed in a flow cytometer (Celesta, mod: BdFACS equipment). As controls, we used cell mixtures of 0, 5, and 30 min of phagocytosis free of antibodies (primary and secondary). Data analysis was performed using the Kaluza software.

### Labeling of recombinant proteins

For experiments using GUVs model, EhVps20, EhVps20(1-173), EhVps32, and EhVps32(1-165) proteins were labeled using Alexa488 (Molecular Probes-Thermo Fisher) accordingly to manufacturer's instructions. The labeled and unlabeled proteins were separated by size exclusion chromatography. The degree of labeling was obtained accordingly to manufacturer's instructions. In all cases, we used 1:5 ratio of labeled: unlabeled proteins to maintain activity.

### Preparation of giant unilamellar vesicles (GUVs)

The lipids 1-palmitoyl-2oleoyl-sn-glycero-3-phosphocholine (POPC), 1-palmitoyl-2-oleoyl-sn-glycero-3-phosphocholine (POPS), cholesterol (chol), 1,2-dioleoyl-sn-glycero-3-phospho-(1′-myo-inositol-3′-phosphate) (PI(3)P), 1,2-dioleoyl-sn-glycero-3-phosphocholine (DOPC), 1,2-dioleoyl-sn-glycero-3-phospho-L-serine (DOPS) and 1,2-dioleoyl-sn-glycero-3-phopho-(1′rac′glycerol) (DOPG) were purchased from Avanti Polar Lipids. In all cases, we added 1,2-Dihexadecanoyl-sn-Glycero-3-Phosphoethanolamine (TexasRed-DHPE) (Molecular Probes) at a concentration of 0.1 mol% in the lipid mixtures for the visualization of the membranes. Giant unilamellar vesicles of different lipid composition were grown using the electroformation method (Angelova and Dimitrov, 1986). Briefly, 10 μl of a 4 mM lipid stock solution in chloroform were spread on indium tin oxide (ITO) coated glasses. The excess of chloroform was eliminated under vacuum at room temperature (RT) for 1h. Then the glasses were assembled with a 2 mm-thick Teflon spacer between them to form the electroformation chamber, which was filled with a 600 mM sucrose solution that matched the osmolarity of the buffer containing the proteins (~650 mOsmol). Finally, an electric AC-field (1.6V, 10 Hz) was applied for 1 h at different temperatures. GUVs were collected and cooled to RT before use.

### Protein binding to the GUVs membrane

GUVs composed of POPC (62 mol%), POPS (10 mol%), chol (25 mol%), and PI(3)P (3 mol%) were grown by electroformation at 60°C. Then, 100 μl of the GUV suspension were placed in an observation chamber and incubated with either rEhVps20 (final concentration 125 nM), rEhVps20 (1-173) (125 nM), rEhVps32 (300 nM) or rEhVps32 (1-165) (300 nM) during 5 min at RT. In all GUVs experiments, the final volume was adjusted to a ratio of 1:1 with the protein buffer (50 mM Tris-HCl, 300 mM NaCl, pH 7.4) or proteins contained in the same buffer. The buffer and all proteins added to the GUVs were osmotically matched to the osmolarity of GUV solution. GUVs were observed with a Leica TCS SP5 confocal microscope. To quantify co-localization of the proteins, the Just Another Co-localization Plugin (JACoP) (Bolte and Cordelieres, 2006) was used in the Image J 1.48 software.

### Reconstitution of ESCRT-III in GUVs

For the reconstitution experiments, 100 μl of GUVs with the same composition as above were placed in an observation chamber and mixed at a final ratio of 1:1 with the proteins or buffer. rEhVps20(1-173) was added to the GUVs to yield a final concentration of 125 nM, after 5 min of incubation at room temperature, rEhVps32 (300 nM) and rEhVps24 (100 nM) were added in that order, separated by 5 min incubation intervals. rEhVps2 (100 nM) was co-incubated with rEhVps24 (100 nM) and added subsequently to rEhVps20(1-173) and rEhVps32. Experiments omitting each protein or altering the order were also performed. Similarly, GUVs were incubated with five rounds of buffer as a negative control. GUVs were analyzed through a Leica TCS SP5 confocal microscope.

### ESCRT-III reactions in negatively charged lipids

GUVs composed of DOPC (100 mol%) or mixed with 5 mol% of PI(3)P or 10 mol% of either DOPS or DOPG were grown by electroformation at RT as described before. GUVs were mixed with rEhVps20(1-173) (125 nM), rEhVps32 (300 nM), and rEhVps24 (100 nM) and incubated for 5 min between de addition of each protein. The final ratio was maintained at 1:1. Percentage of GUVs with ILVs was measured in 100 randomly observed GUVs with a diameter in the range of 25 to 30 μm. The mean and standard error was obtained from three independent experiments.

### Generation of EhVps20 and EhVps24 knock down trophozoites

The first 444 bp from the 5′ end of the *Ehvps20* gene were PCR-amplified and cloned into the *pJET1*.*2/blunt* plasmid and then, subcloned into *pSAP2/Gunma* plasmid, downstream of the 5' upstream segment of the *EhAp-A* gene, using a 5′ *StuI* site and a 3′ *SacI* site with the following primers: forward, 5′-CCAAGGCCTATGTTAAATCGATTCATTGGAAAGA-3′; reverse, 5′-CACGAGCTCTCTTGTGATAAAATGTCACCAAATT-3′ (*StuI* and *SacI* restriction sites are underlined, respectively). Trophozoites of clone G3 were transfected as described (Bracha et al., 2006). Briefly, G3 trophozoites were cultured in 35-mm Petri dishes and transfected with 20 μg of each plasmid: *pSAP2/GunmaEhVps20* (1–444 bp) or *pSAP/Gunma*, using SuperFect (Qiagen) reagent. The transfected parasites were incubated for 24 h at 37°C and then, EhVps20 silencing was confirmed by western blot analysis, immunofluorescence assays and rate of erythrophagocytosis. In the case of the *Ehvps24* gene, the first 426 bp were PCR-amplified using the following primers: forward, 5′-GAGAGGCCTATGGGCAACCTTAATAGCCA-3′; reverse, 5′-CCTGAGCTCTTGTTCATACAATGAATCAATCTCC-3′ (*StuI* and *SacI* restriction sites are underlined, respectively). The products were cloned and treated following the protocol previously described.

## Ethics statement

The Centre for Research and Advanced Studies (CINVESTAV) fulfill the standard of the Mexican Official Norm (NOM-062-ZOO-1999) “Technical Specifications for the Care and Use of Laboratory Animals” based on the Guide for the Care and Use of Laboratory Animals “The Guide,” 2011, NRC, USA with the Federal Register Number BOO.02.03.02.01.908, awarded by the National Health Service, Food Safety and Quality (SENASICA) belong to the Animal Health Office of the Secretary of Agriculture, Livestock, Rural Development, Fisheries and Food (SAGARPA), an organization that verifies the state compliance of such NOM in Mexico. The Institutional Animal Care and Use Committee (IACUC/ethics committee) from CINVESTAV as the regulatory office for the approval of research protocols, involving the use of laboratory animals and in fulfillment of the Mexican Official Norm, has reviewed and approved all animal experiments (Protocol Number 0505-12, CICUAL 001).

## Author contributions

YA-P performed the experiments with GUVS, discussed experiments and results, wrote the manuscript; RK directed experiments with GUVS, discussed experiments and results; RJ-R performed the immunofluorescence and flow cytometry experiments, discussed experiments and results; GG-R performed experiments with *E. histolytica*, obtained mutants; RL directed the project related to GUVs, reviewed the manuscript, discussed strategies and experiments; RD directed the project related to GUVS, proposed and directed experiments, reviewed the manuscript; EO directed the project related to the biology of *E. histolytica*, proposed and directed experiments and wrote and reviewed the manuscript.

### Conflict of interest statement

The authors declare that the research was conducted in the absence of any commercial or financial relationships that could be construed as a potential conflict of interest.

## Supplementary material

Figure S1 Effect of different combinations of ESCRT-III proteins in GUVs. GUVs prepared from POPC (62 mol%), POPS (10 mol%), chol (25 mol%), PI(3)P (3 mol%) and TR-DHPE (0.1 mol%) were incubated with different combinations of ESCRT-III proteins indicated at left during 5 min. GUVs were analyzed through confocal microscope. Scale bar: 20 μm. Accession numbers/ID for proteins and genes mentioned in the text. EhADH (Q9U7F6/EHI_181220), EhCp112 (QPI7F7/EHI_181230), EhVps32 (C4M1A5/EHI_169820), EhVps2 (C4LZV3/EHI_194400), EhVps20 (C4M7T5/EHI_114790), EhVps24 (C4M2Y2/EHI_048690), EhVps4 (C4LYN8/EHI_118900), Gal/Gal lectin (C4lTM0/EHI_012270).
