# Supplementary figures and images for: The Conserved ESCRT-III Machinery Participates in the Phagocytosis of Entamoeba histolytica
Source: Front Cell Infect Microbiol. 2018 Mar 1;8:53. doi: 10.3389/fcimb.2018.00053 (PMC5838018; doi:10.3389/fcimb.2018.00053)

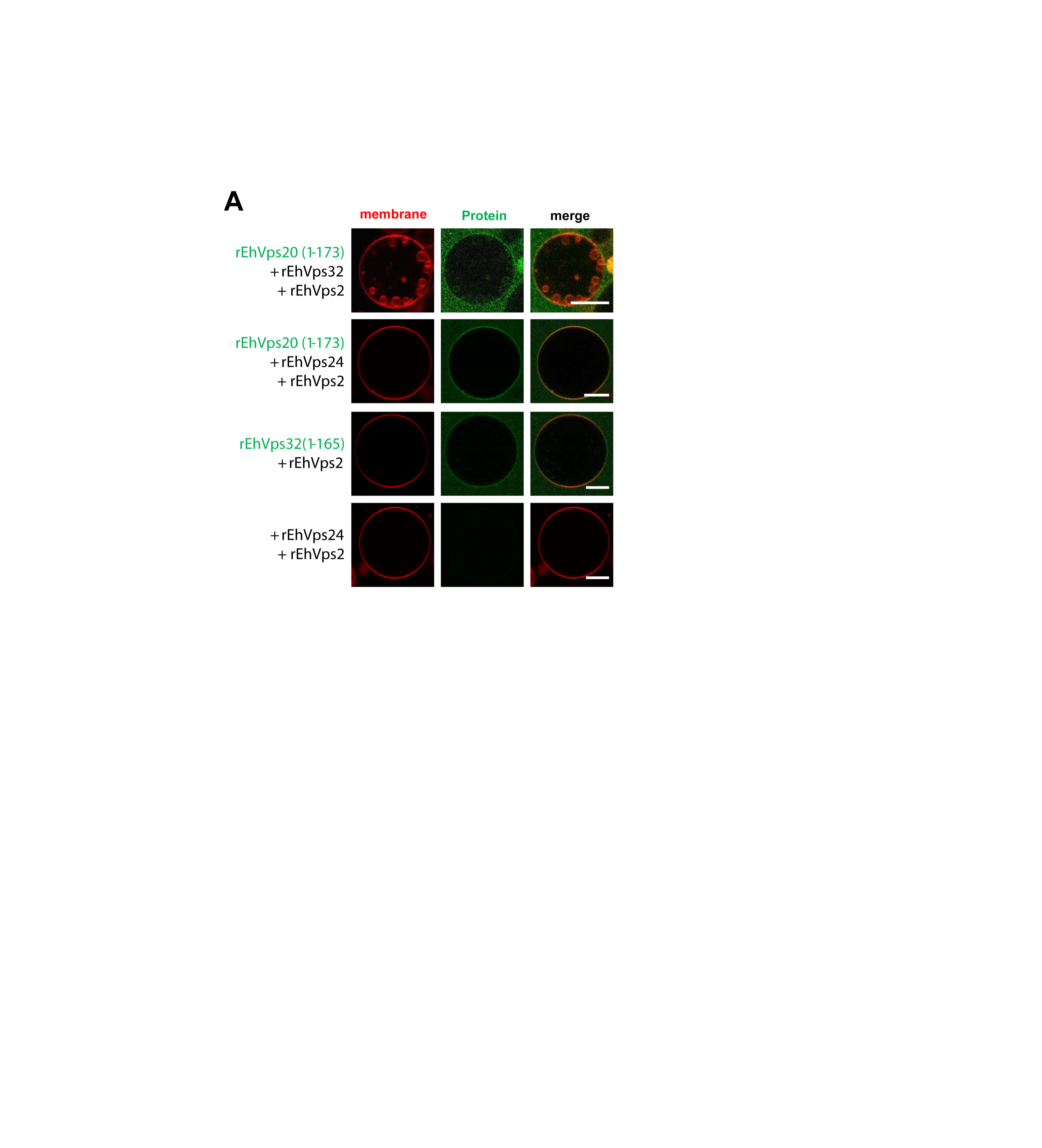

Supplement: Figure S1 — Effect of different combinations of ESCRT-III proteins in GUVs. GUVs prepared from POPC (62 mol%), POPS (10 mol%), chol (25 mol%), PI(3)P (3 mol%) and TR-DHPE (0.1 mol%) were incubated with different combinations of ESCRT-III proteins indicated at left during 5 min. GUVs were analyzed through confocal microscope. Scale bar: 20 μm. Accession numbers/ID for proteins and genes mentioned in the text. EhADH (Q9U7F6/EHI_181220), EhCp112 (QPI7F7/EHI_181230), EhVps32 (C4M1A5/EHI_169820), EhVps2 (C4LZV3/EHI_194400), EhVps20 (C4M7T5/EHI_114790), EhVps24 (C4M2Y2/EHI_048690), EhVps4 (C4LYN8/EHI_118900), Gal/Gal lectin (C4lTM0/EHI_012270). [file Image1.TIF]
